# Current Approaches to Following Up Women and Newborns After Discharge From Childbirth Facilities: A Scoping Review

**DOI:** 10.9745/GHSP-D-23-00377

**Published:** 2024-04-29

**Authors:** Maxine Pepper, Oona M.R. Campbell, Susannah L. Woodd

**Affiliations:** a Faculty of Epidemiology and Population Health, London School of Hygiene & Tropical Medicine, London, United Kingdom.

## Abstract

**Introduction:**

The postpartum period is critical for the health and well-being of women and newborns, but there is limited research on the most effective methods of post-childbirth follow-up. This scoping review synthesizes evidence from high-, middle-, and low-income countries on approaches to following up individuals after discharge from childbirth facilities.

**Methods:**

Using a systematic search in Ovid MEDLINE, we identified quantitative studies describing post-discharge follow-up methods deployed up to 12 months postpartum. We searched for English-language, peer-reviewed articles published between January 1, 2007 and November 2, 2022, with search terms covering 2 broad areas: “postpartum/postnatal period” and “surveillance.” We single-screened titles and abstracts and double-extracted all included articles, recording study design and location, population, health outcome, method, timing and frequency of data collection, and percentage of study participants reached.

**Results:**

We identified 1,654 records, of which 31 studies were included. Eight studies used in-person visits to follow up participants, 10 used telephone calls, 7 used self-administered questionnaires, and 6 used multiple methods. Across studies, the minimum length of follow-up was 1 week after delivery, and up to 4 contacts were made within the first year after delivery. Follow-up (response) rates ranged from 23% to100%. Postpartum infection was the most common outcome investigated. Other outcomes included maternal (ill-)health, neonatal (ill-)health and growth, maternal mental health and well-being, care-giving/-seeking behaviors, and knowledge and intentions.

**Conclusion:**

Our scoping review identified multiple follow-up methods after discharge, ranging from home visits to self-administered electronic questionnaires, which could be implemented with high response rates. The studies demonstrated that post-discharge follow-up of women and newborns was feasible, well received, and important for identifying postpartum illness or complications that would otherwise be missed. Therefore, the identified methods have the potential to become an important component of fostering a continuum of care and measuring and addressing postpartum morbidity.

## Abstract (precis)

This review found that follow-up after childbirth can be implemented using a variety of methods after discharge with high response rates in most studies and have the potential to be integrated into routine health care approaches.

## Teaser

**Key Findings:**

- Intensified post-childbirth follow-up creates opportunities to detect childbirth-related complications and to offer support. This scoping review describes current approaches to following women and newborns up to 1 year after they leave childbirth facilities.
- Follow-up was conducted via in-person visits, telephone calls, self-administered questionnaires, or a combination—with high response rates in most studies.
- The included studies illustrated the range of methods available and demonstrated that post-discharge follow-up of women and newborns was feasible, well received, and important for identifying cases of postpartum illness or complication that would otherwise be missed.

**Key Implications:**

- Those using follow-up methods should report information on the cost of and resources needed for follow-up. More robust evaluations should examine outcomes beyond infection and assess validity and cost-effectiveness.
- With a variety of methods to choose from, health care facilities and public health authorities should consider integrating post-discharge follow-up into routine health care approaches. Recognizing the need for further testing and local prioritization, the follow-up methods identified could potentially become an essential component of fostering a continuum of care and measuring and addressing postpartum morbidity.

## INTRODUCTION

The first weeks and months after childbirth are critical periods for women and newborns. The risk of maternal and neonatal death is highest around the time of delivery, but mothers and newborns continue to be at increased risk of morbidity and mortality in the postpartum period (i.e., the first 42 days after childbirth).^1^^–^^4^ Consequently, various global initiatives have emphasized the importance of providing skilled care in the postpartum period.^5^^–^^8^ There are also various childbirth-related complications that arise and persist after the postpartum period, including, but not limited to, dyspareunia (estimated prevalence: 35%), low back pain (32%), urinary incontinence (8%–31%), and depression (11%–17%).^9^ Moreover, women all over the world express needs for psychological support, reassurance for minor “everyday” concerns, and information on how to care for their baby.^10^^–^^13^ This highlights the need to establish good systems of support and follow-up care that reach women and newborns after they leave childbirth facilities.

Historically, in high-income countries, long lengths of stay allowed postpartum care to be delivered within childbirth facilities. However, women are now discharged much earlier in many high-income countries.^14^ Across 30 low- and middle-income countries, the length of stay after childbirth was also found to be short, with an average as low as 1.3 days in some countries.^15^ Recognizing the importance of postpartum care, the 2022 World Health Organization Guidelines^8^ recommend good-quality postnatal care in the first days after childbirth and the need for at least 3 postnatal contacts in the 6 weeks postpartum. However, a study across 33 sub-Saharan African countries found one-third of women did not receive a single health check between delivery and health facility discharge.^16^ Similarly, coverage data from over 90 countries show levels of postnatal care (at 60% for mothers, 41% for babies) are below those for antenatal (85%) or institutional delivery (73%).^17^ Consequently, there is limited opportunity for health care providers to identify and address the health and well-being needs of women and newborns after childbirth.

Gaps in the provision of care after discharge from childbirth facilities also limit our understanding of the epidemiology of postpartum morbidity^18^ and can mean that health care providers are less aware of its burden in the community. A review on the incidence of maternal peripartum infections concluded that many infections were missed because only 20%–43% (depending on the condition under investigation) of included studies specified follow-up beyond discharge.^19^ The European Centre for Disease Prevention and Control reported that in 2016, 85% of surgical site infections (SSIs) after cesarean delivery were diagnosed after discharge^20^ and that countries with more intensive surveillance methods identified more infections.^21^ Moreover, restricting postnatal services to the first 6 weeks postpartum has been suggested to contribute to the neglect of the medium- and long-term labor- and childbirth-related complications.^9^ The paucity of prevalence estimates coincides with the absence of high-quality clinical guidelines for some of these conditions, especially in many low- and middle-income countries. Understanding the true burden of morbidity is an important cornerstone of developing effective clinical care guidelines. Therefore, improving measurement of post-childbirth health outcomes is not just of research interest but also a prerequisite for improving care.

Intensified follow-up of women and newborns after discharge from childbirth facilities has the potential to foster a continuum of care, identify and address problems with service provision, enhance quality improvement efforts, produce evidence on the prevalence of labor- and childbirth-related complications, and support advocacy for improved maternal health care.^22^ However, program implementors need to be cognizant of contextual and resource constraints that limit their choices regarding frequency and mode of follow-up contact. Expanding traditional in-person postnatal clinic visits may not be feasible in all settings, especially where transport costs are high. For low-resource settings, follow-up methods should ideally be low cost, efficient, valid, and not rely on individual electronic records or functioning postal systems. Program reports offer some examples of alternative modes of follow-up. The Safe Deliveries program tested home visits by community health volunteers with mothers of small babies in Zanzibar.^23^ These additional contacts were reported to positively affect attendance at routine check-up appointments. The Noora Health program in India used WhatsApp to engage mothers remotely after discharge, thereby addressing a gap in follow-up created by the COVID-19 pandemic.^24^ These examples illustrate some methods that can be used to follow women and newborns after discharge. Nevertheless, limited research has been conducted to describe and compare these methods, and we were unable to find a systematic review of this topic.

Intensified follow-up of women and newborns after discharge from childbirth facilities has the potential to foster a continuum of care, identify and address problems with service provision, and enhance quality improvement efforts.

This scoping review aims to identify and synthesize evidence from high-, middle- and low-income countries on the various methods deployed to follow-up women and newborns after discharge from childbirth facilities. The specific objectives are to identify and describe the methods used for follow-up, describe the range and timing of outcomes studied, and report on follow-up (response) rates. We focus on methods that have the potential to be employed routinely and at a large scale. This review offers an overview of the diversity of methods available for post-discharge follow-up that we hope will encourage more research to test and evaluate these methods further. By mapping the existing literature and identifying gaps, challenges, and opportunities, our review provides a resource for health care providers, program managers, and policymakers who hope to gain a deeper understanding of the postpartum experience of women and newborns and provide them with services.

## METHODS

We followed the PRISMA Extension for Scoping Reviews reporting guidelines.^25^

### Eligibility Criteria

The review was designed to identify peer-reviewed publications on methods that could be used to follow parents and newborns after discharge and in the postpartum period, up to 12 months post-delivery. To describe the diversity of follow-up methods available, we searched for publications ranging from follow-up in the context of research studies to follow-up in the context of active surveillance systems (i.e., systems that actively contact the population to seek information about health conditions^26^). Follow-up methods were of interest if they aimed to contact every member of the study population and to screen those contacted systematically for the outcome(s) of interest. Eligible studies needed to (1) have been peer reviewed, (2) followed up parents or newborns after discharge post-birth, (3) sought information from every member of the study population, and (4) have specified the proportion of the study population retained at follow-up.

We excluded (1) review articles, study protocols, conference abstracts, and commentaries; (2) qualitative work; (3) studies with a first follow-up contact more than 1 year after birth; and (4) studies reporting mortality outcomes only (because data sources and methods for mortality surveillance differ from those of morbidity^27^^,^^28^). Studies were also excluded if follow-up was restricted to in-patients, findings were primarily derived from secondary analyses based on data from existing surveillance networks (rather than providing original insights into the proportion of the study population retained in follow-up), or the primary aim of follow-up was to describe the success of an intervention (rather than to report on the health status of the study population). By excluding evaluations of trials (where the intervention was unrelated to follow-up/surveillance), we hoped to increase the relevance of our findings to routine health care approaches. Intervention trials are unusual in that they are often highly resourced, make extraordinary efforts and multiple contacts to achieve complete follow-up, and have a select group of study subjects who are willing to participate in an intervention trial.

Where multiple articles reported data derived from the same surveillance project, we only included the article with the original description of the follow-up method and excluded others unless they offered additional insights into the success of the approach (e.g., by specifying response rates for subgroups or for different time periods).

### Information Sources and Search Strategy

We searched the Ovid MEDLINE database, restricting our search to English-language articles published between March 1, 2007, and November 2, 2022. The search strategy combined search terms related to the focus domain (surveillance) and the time period of interest (postpartum/postnatal period) with the Boolean operator AND. We included both free text and medical subject headings (the Supplement includes the full search strategy and terms).

We also searched the reference lists of articles that were excluded because they reported secondary analyses from existing parent cohort/surveillance systems to identify and include the original description of the follow-up method.

### Screening and Data Extraction

Titles and abstracts were single screened by SW (1,194 articles) and MP (460 articles). All included articles were double abstracted, with OC as a third reviewer when needed.

Data items extracted included the study location, study population, health outcome(s) of interest, length of postpartum follow-up, method of data collection (including timing and frequency of attempts to reach women), and percentage of study participants reached (response/follow-up rates). We compiled the extracted information in a Microsoft Excel table.

### Data Synthesis

Using an inductive approach, we created categories of follow-up methods and assigned studies to these. Within the table presented, we grouped studies that reported findings from the same surveillance system. While these studies shared some of their methods, they reported unique response rates and were therefore counted as separate studies. Lastly, we classified the countries in which the studies took place as least-developed, low-income, middle-income (combining lower- and upper-middle-income), and high-income countries based on the Development Assistance Committee 2022/2023 list of official development assistance recipients.^29^

## RESULTS

We identified 1,654 articles via the search strategy and 3 more by searching for the original description of the follow-up method mentioned in identified articles (Figure). Of these, we included 31 studies providing data on post-discharge follow-up methods (Table).^30^^–^^60^ For some follow-up approaches, we identified multiple relevant studies; 2 studies were based on the Norwegian Mother and Child Cohort Study (MoBa),^48^^,^^49^ 2 studies on the MINA-Brazil Cohort,^56^^,^^57^ and 2 studies on the Pregnancy Risk Assessment Monitoring System (PRAMS) in the United States.^50^^,^^51^ A study in Puerto Rico^45^ used some of the PRAMS methods, but we considered it to be distinct from the PRAMS studies in the United States and kept it separate. The Table lists the 31 studies with a description of geographic location, design, population, sample size, study outcome, and follow-up in terms of response rate, method, timing, and persistence.

**FIGURE:**
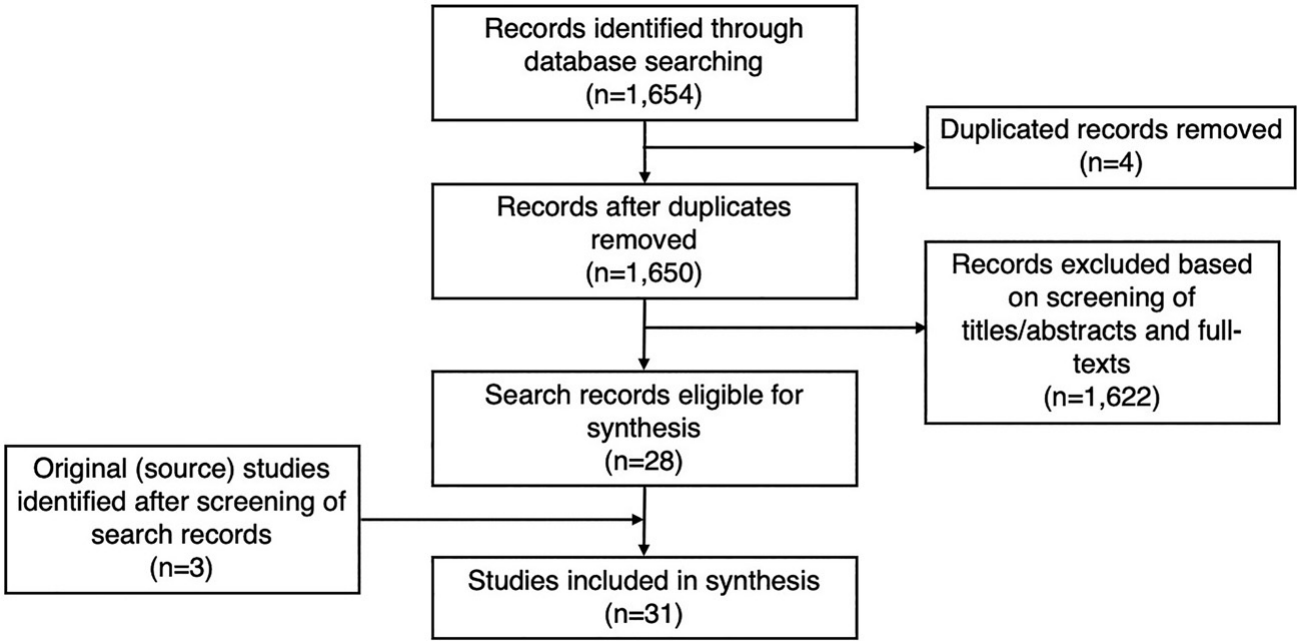
Process of Selecting Studies on Follow-Up of Women and Newborns After Discharge From Childbirth Facilities

### Study Settings, Study Designs, and Study Populations

Of the 31 studies, 7 were from the United States,^37^^,^^40^^–^^42^^,^^50^^,^^51^^,^^53^ 10 from Europe,^34^^,^^36^^,^^38^^,^^46^^,^^48^^,^^49^^,^^52^^,^^55^^,^^58^^,^^60^ 5 from Latin America and the Caribbean,^39^^,^^43^^,^^45^^,^^56^^,^^57^ 4 from Asia,^32^^,^^33^^,^^35^^,^^54^ 3 from sub-Saharan Africa.^31^^,^^44^^,^^47^ Two covered multiple countries (1 with sites in sub-Saharan Africa and Latin America^59^ and the second in sub-Saharan Africa and South Asia^30^). There were 2 cross-sectional studies (1 repeated cross-sectional),^52^^,^^55^ and 2 controlled trials (1 cluster-randomized).^32^^,^^53^ The remaining 27 studies were prospective cohorts. Sample sizes ranged from 193^41^ to 347,363.^51^

Study participants were either recruited during pregnancy or after delivery. Seventeen studies sampled study participants with specific characteristics, with 11 studies focusing on individuals who had a cesarean delivery.^34^^–^^36^^,^^39^^,^^41^^,^^43^^,^^44^^,^^46^^,^^55^^,^^58^^,^^60^ Other characteristics of interest included testing positive for COVID-19 in the hospital,^42^ experiencing no pelvic pain during pregnancy,^49^ having pregnancy-related hypertension,^53^ losing a baby after birth,^51^ belonging to the rural-to-urban floating population,^54^ and being HIV-positive.^37^ Two studies also contacted fathers,^45^^,^^48^ but these contacts did not take place in the first year after delivery.

### Outcomes Measured

The most common outcome, measured by 13 studies, was postpartum infection (1 included newborn infection).^34^^–^^36^^,^^38^^,^^39^^,^^41^^,^^43^^,^^44^^,^^46^^,^^47^^,^^55^^,^^58^^,^^60^ Follow-up usually lasted up to 1 month after delivery, and the majority (11) of these studies were conducted among individuals with cesarean delivery. Multiple studies reported that infections were often diagnosed after discharge.

Various other outcomes were assessed across the other 18 studies, with some studies measuring multiple outcomes. Outcomes included maternal health/ill-health^30^^,^^33^^,^^40^^,^^42^^,^^45^^,^^48^^,^^49^^,^^52^; neonatal health/ill-health and growth^32^^,^^45^^,^^48^^,^^49^^,^^56^^,^^57^; maternal mental health, well-being, and substance abuse^33^^,^^37^^,^^50^^,^^51^^,^^54^; caregiving/care-seeking behaviors^31^^,^^45^^,^^50^^,^^51^^,^^53^^,^^59^; and knowledge and intentions.^31^^,^^51^^,^^52^ Two of these studies measured mortality in addition to morbidity.^30^^,^^32^

### Data Collection Methods, Lengths of Follow-Up, and Response Rates

The 31 studies used a variety of methods to follow up individuals in the postpartum period; 8 used in-person visits,^30^^–^^38^ 10 studies used telephone calls primarily,^38^^–^^47^ 7 used self-administered questionnaires,^48^^–^^54^ and 6 used a combination of multiple methods.^55^^–^^60^ Minimum length of follow-up was up to 1 week after delivery^40^ (maximum length of follow-up was restricted to 1 year). The follow-up rates ranged from 23%^52^ to 100%.^60^ Within the first year after delivery, some studies had as many as 4 contacts with study participants. If studies reported individual response rates for each time point, response rates tended to decline over time, except for a study in Brazil.^43^ Some studies (mainly those using telephone calls and self-administered questionnaires) reported the number of attempts of establishing contact at each time point (up to 5 mailings and 15 call attempts^50^^,^^51^). Only 1 study from Tanzania provided data on time and costs, reporting that phone interviews lasted 3–5 minutes with an average cost of US$0.50.^44^

#### In-Person Visits

Eight studies conducted in-person visits, which took place in either the participant’s home (5 studies), a health care facility (2 studies), or an unspecified location (1 study).

Across the 5 studies conducting home visits (Table, Section 1.1), the response rates ranged from 73% to 96%. The highest response rate was recorded in a study in Bangladesh that had trained interviewers conduct home interviews to ask about maternal morbidity (at 3 months) and depressive symptoms (at 6 months).^33^ The lowest response rate was observed in a study on neonatal illness and survival in Bangladesh. Household visits by community health workers were scheduled for days 2, 5, 8, and 28 after delivery, but only 73% of households were visited at least once.^32^ Most (4) studies took place in least-developed or middle-income countries and investigated broad outcome domains. Home visits were conducted by research staff (3 studies) or community health staff (1 study used community health workers, and 1 study used community nurses).

The 2 studies using clinic visits primarily (Table, Section 1.2) achieved response rates of 77% and 86%. Both studies investigated SSIs after cesarean delivery (up to 30 days after delivery) and used microbiological methods for confirmation. Clinical assessments were conducted by nurses, surgeons, or gynecologists. The response rate of 86% was recorded for clinic visits on day 15 in a study in Cambodia^35^; however, 9% of those reached did not actually return to the clinic and had to be followed up by telephone. The authors also reported that response rates for visits on day 30 decreased to 80%.

The Surveillance Monitoring for Antiretroviral Toxicities study (Table, Section 1.3) did not specify the location of the visits but conducted follow-up through in-person structured interviews.^37^ Response rates were 98% at 1 week and 60% at 12 months after delivery.

#### Telephone Calls

Ten studies followed up individuals using telephone calls (Table, Section 2), with most (7) studies measuring infection. Only 3 studies measured broader outcome domains. The response rates ranged from 36% to 97%. The highest response rate was recorded in an Italian study that had 2 physicians call participants up to day 30 after delivery and assess them for postpartum infection.^38^ In contrast, among mothers who tested positive for COVID-19 as they delivered in a U.S. hospital, only 36% responded to phone calls enquiring about their well-being up to 2 weeks after discharge.^42^ The majority (7) of the studies reported that participants were called multiple times if they could not be reached, with up to 5 attempts at each contact time point.

#### Self-Administered Questionnaires

Of the 7 studies using self-administered questionnaires, 5 dispatched questionnaires by post and 2 electronically (using email or WeChat, and text message). One took place in China and the rest in high-income countries.

For the 5 studies using postal questionnaires (Table, Section 3.1), response rates ranged from 23% to 85%. In Norway, 80% of participants responded to a questionnaire about maternal and child health outcomes, which was sent 6 months after delivery.^48^ Response rates were even higher (85%) in a subgroup of the study population that did not experience pelvic pain during pregnancy.^49^ In a study on perineal morbidity in the United Kingdom, the response rate to a postal questionnaire was as low as 23% at 12 months after delivery.^52^ The other 2 studies also investigated broad outcome domains (maternal behaviors, attitudes, and experiences). Some studies specified that they sent out reminders or followed up non-responders using other methods (for example, telephone calls).

Electronic questionnaires were used in 2 instances (Table, Section 3.2) to record blood pressure readings (via text messages)^53^ and to assess mental health (questionnaire sent via email or WeChat).^54^ The response rates were 92% for the text messages in the United States (day 10) and 81% for the questionnaire distributed via email or WeChat in China (week 6). Both studies sent reminders and focused on a study population with special characteristics (having pregnancy-related hypertension or belonging to a rural-to-urban floating population).

#### Combinations of Methods

Six studies followed up women using a combination of the previously described methods (Table, Section 4); 4 used a combination of in-person visits and telephone calls,^56^^–^^59^ 1 used a combination of telephone calls and self-administered questionnaires,^55^ and 1 used a combination of all the 3 methods.^60^ In addition, 3 studies also used electronic hospital record linkage.^55^^–^^57^ Across these 6 studies, response rates ranged from 47% to 100%. Complete (100%) follow-up of up to day 30 was achieved by a Norwegian study on SSI among patients with a cesarean section.^60^ This study offered wound inspection in the hospital, instructed participants to monitor symptoms and contact the hospital if needed, and followed them up with postal questionnaires and telephone calls. In comparison, response rates were lower in a United Kingdom study identifying cesarean SSI cases using a combination of electronic record screening, telephone calls, and text messages.^55^ The study was conducted at 4 time points, with response rates ranging from 47% to 68%. As the accuracy of the telephone numbers improved, response rates were reported to increase.

### Follow-up Methods by Country Setting and by Study Population

The majority (17) of the 31 studies were conducted in high-income countries. Seven took place in middle-income countries,^31^^,^^36^^,^^39^^,^^43^^,^^54^^,^^56^^,^^57^ 5 in least-developed countries,^32^^,^^33^^,^^35^^,^^44^^,^^47^ and 2 in multiple settings including both least-developed and middle-income countries.^30^^,^^59^ Among the studies in least-developed countries, in-person visits were common (3 of 5 studies), and no study tested self-administered questionnaires. In comparison, only 2 high-income studies used in-person visits, while 6 studies distributed self-administered questionnaires. In middle-income countries, all 4 follow-up methods were implemented. The response ranged from 73%^32^ to 96%^33^ in least-developed countries, from 63%^57^ to 92%^39^ in middle-income countries, and from 23%^52^ to 100%^60^ in high-income countries. In the studies including multiple countries, response rates were 91%^30^ and 98%.^59^ Some studies (11) included only women who had a cesarean delivery (no study focused on women with vaginal delivery exclusively). To contact women after a cesarean delivery, all follow-up methods were used except for self-administered questionnaires. Response rates ranged from 47%^55^ to 100%.^60^

## DISCUSSION

Using a systematic search strategy, we identified 31 studies describing methods to follow individuals for up to 1 year postpartum after they left childbirth facilities. The follow-up methods were categorized as in-person visits, telephone calls, self-administered questionnaires, or a combination of these. In-person visits were most commonly implemented in least-developed countries, whereas self-administered questionnaires were nearly exclusively implemented in high-income countries. For each of the 4 follow-up methods, we observed a range of response rates, with most methods reaching the majority of participants. Compared to studies using a single method (i.e., in-person visits, telephone calls, or self-administered questionnaires), those using a combination of methods did not have higher response rates. There was also no clear link between country setting and response rates. To increase response rates, some authors reported using reminders and mixing methods to reach non-responders. Overall, our findings suggest that all methods—in-person visits, telephone calls, self-administered questionnaires, or a combination—can be successfully employed to reach participants after discharge from childbirth facilities.

Our findings suggest that in-person visits, telephone calls, self-administered questionnaires, or a combination of these can be successfully used to reach participants after discharge from childbirth facilities.

In addition to feasibility, the high response rates suggest that all these methods can be implemented in a manner that is acceptable and well received by individuals in the postpartum period. Recent qualitative work demonstrated that women appreciate having phone interviews about their childbirth experience and are motivated by a desire to improve facility-based care.^61^ While researchers need to be cognizant of acceptability in all cases, the literature to date certainly does not suggest that women are reluctant to speak on the telephone.

The identified studies investigated a broad range of outcomes, and the operationalization of the follow-up methods in terms of timing, frequency, person implementing, and persistence differed accordingly. For example, length of follow-up for postpartum infection was usually up to 1 month after delivery, in line with the definition of SSI by the U.S. Centers for Disease Control and Prevention.^62^ All follow-up methods were implemented for different target outcomes. More specific outcomes, such as postpartum infection, tended to be measured through clinic visits, telephone calls, or a combination of methods. This suggests that phone calls are a suitable tool for investigating clearly defined outcomes using a set of standardized questions. Clinic visits allow for more elaborate outcome assessments, especially those requiring laboratory or clinical diagnostic verification (for example, relying on bacterial cultures). Broader outcome domains, such as maternal morbidity or mental health, were most investigated through home visits, self-administered questionnaires, or a combination of methods. Consequently, the choice of method and timing of follow-up contact seems to depend on the objective and outcome under investigation. To cover the entire spectrum of postpartum conditions, consideration should be given to using a combination of methods where this ensures greater validity, feasibility, and cost-effectiveness.

The study setting is another factor that is likely to influence the choice and success of follow-up methods. For example, literacy levels,^63^ reliability of postal service, and internet access may be key concerns for the implementation of self-administered questionnaires. This could explain why we observed self-administered questionnaires were mostly implemented in high-income countries. In the future, phone and Internet-based follow-up methods have the potential to become much more widespread, given that phone ownership and Internet access are increasing rapidly around the world, especially among young people of reproductive age.^64^ However, the persisting between- and within-country inequalities in (smart-)phone ownership could hinder the rollout of these methods in some settings.^64^^,^^65^ For clinic visits, key contextual factors include ease and cost of returning to clinics. Across the identified studies, we did not find evidence that response rates differ across least-developed, middle-income, and high-income countries. Currently, more research on follow-up methods is conducted in high-income settings, although we expect that the impact of increased follow-up in terms of case identification and linkage to care will be even greater in settings where underlying morbidity is higher.

In terms of measurement, multiple studies on SSI reported that the described methods identified post-discharge cases that would have been missed otherwise, thereby making an important contribution to estimating the incidence of postpartum morbidity more accurately. However, other authors have previously criticized post-discharge surveillance methods, stating that the validity and reliability are rarely evaluated.^66^ Indeed, only 1 of the included studies evaluated the described method (telephone interviews as a potential diagnostic tool for SSI) against a gold standard (clinical reviews), finding that telephone interviews had a sensitivity of 71%, a specificity of 100%, a positive predictive value of 100%, and a negative predictive value of 96%.^15^ Earlier research reported a poor correlation between patients’ self-assessment of SSI and diagnosis by experienced infection control nurses (positive predictive value: 29%, negative predictive value: 98%).^67^ This suggests there is a need to better understand the validity of the described follow-up methods. However, these methods seem to be successful at identifying cases that would go unnoticed if no alternative postpartum surveillance systems were in place.

In addition to having high response rates and good sensitivity and specificity, the ideal follow-up method has been described as being cost effective and not time consuming.^68^ The included studies rarely reported on these parameters, and only 1 study reported actual costs.^25^ Other authors have previously reported that phone interviews can be very labor intensive.^69^ In the future, better reporting on such information would be desirable. Generally, the cost of follow-up is likely to increase with the level of data collector effort (number of contacts, travel time to site of in-person visit, interviewer- rather than self-administered questionnaires). These additional costs of the interventions may, however, be balanced or mitigated by the reductions in the costs and burdens of unidentified and untreated morbidity to the health system, family, and society. A more holistic evaluation of surveillance and follow-up methods should also consider exploring other dimensions of success beyond response rates, including but not limited to successful referral and treatment of those contacted.

Our search has focused on follow-up approaches that have the potential to become integrated into routine health care approaches. We excluded studies on passive surveillance approaches which do not aim to contact all study participants, for example surveillance via linkage of electronic health records. The ability of such systems to capture all cases cannot be assumed but is dependent on good electronic record-keeping, access to services, and health care-seeking behavior of individuals. Nevertheless, we acknowledge that the advent of electronic record linkage in settings where nearly all women have unfettered access to health services means such study designs are increasingly common. In some places, surveillance systems based on electronic record linkage are already implemented in the postpartum period,^70^^,^^71^ and such systems and other novel methods (for example, mobile apps^72^) are likely to become more important in the future. We also excluded studies with follow-up methods specifically designed to evaluate the success of an intervention. Intervention trials can sometimes invest extensive resources into follow-up, with many efforts to contact women. For example, we excluded a Cambodian supplementation trial in which salt intake in women (up to 6 months after birth) was evaluated via household salt disappearance studies, 12-hour observation periods, and urinary samples.^73^ Such methods seem unsuitable for large-scale rollout. In addition, study participants in intervention trials tend to differ systematically from nonparticipants,^74^^–^^76^ so response rates recorded in intervention trials are unlikely to be generalizable to a wider population. By excluding intervention trials, we hoped to present a more realistic picture of methods that have the potential to be used regularly within facilities as routine health care approaches. Nonetheless, the identified methods were still often tested in a research context, and their integration into routine health services may hold additional challenges.

Part of the rationale for following up postpartum women and newborns is that this is a period where they may need additional care. However, some subgroups may be particularly at high risk and could benefit from the implementation of targeted postpartum follow-up systems. These include but are not limited to preterm and low birth weight babies, twins, women with a stillbirth, and women with complicated “near-miss” deliveries. Improving follow-up care for high-risk groups is likely to be very impactful in terms of detecting and treating morbidity and preventing mortality. However, our search strategy was not designed to find work on these groups specifically, except for women who had a cesarean delivery. The follow-up methods identified by our review could potentially be used within these high-risk subgroups, and there are examples of studies using home visits,^77^ video consultations,^78^ telephone calls,^79^ and email questionnaires^80^ to do so. Nevertheless, the identified methods would need to be carefully adapted to meet the specific follow-up needs of these high-risk subgroups in terms of relevant outcomes, and timing and frequency of contact.

### Strengths and Weaknesses

Our review of approaches to following up women and newborns after discharge has been informed by a systematic search without any geographic exclusion criteria. Therefore, we are able to describe approaches used in a wide range of settings.

Our search strategy was not exhaustive, and it is possible that more examples of follow-up methods could have been identified by searching other databases, expanding the list of search terms, and lifting exclusion criteria based on year of publication or language. We did not look at gray literature and may have missed programmatic experience that was not in peer-reviewed journals. Moreover, the double extraction was most frequently discordant on whether to exclude studies because surveillance was part of evaluating an intervention. While we resolved all these discrepancies, we acknowledge that these decisions were potentially nonreplicable.

From the articles included, we extracted response but not participation rates, meaning that it is not possible to infer the percentage of participants reached among those eligible. Consequently, the reach of some approaches may be more limited than the response rates suggest.

## RECOMMENDATIONS

Short stays within childbirth facilities, suboptimal uptake of routine postnatal care check-ups, and a strong focus on the first 6 weeks postpartum limit opportunities to detect childbirth-related complications and support women. Our scoping review illustrates that there is a diverse set of approaches available to follow up women and newborns after they leave childbirth facilities, all of which can be implemented with high response rates. The studies presented demonstrate that post-childbirth follow-up is feasible, well received, and important for identifying cases that would otherwise be missed. This should encourage health care facilities and public health authorities to consider integrating post-discharge follow-up as part of routine health care approaches, particularly if these can be provided cost effectively. In a research context, these approaches might be used to study complications emerging after discharge—an important step toward addressing the historic neglect of postpartum conditions.

The studies presented demonstrate that post-childbirth follow-up is feasible, well received, and important for identifying cases that would otherwise be missed.

If a clinical assessment is needed to validly measure an outcome of interest, the main options available are either home visits conducted by clinicians (e.g., nurses) or asking individuals to travel to clinics for assessment. Both approaches incur time and travel costs, and clinical assessment costs. In contrast, for outcomes that can be self-reported, telephone calls present a promising method to replace more traditional modes of delivering follow-up care, such as home or clinic visits, thereby reducing time and monetary costs to providers or users. In settings with widespread Internet access, electronic questionnaires could also be implemented. Phone and Internet-based methods may be used for initial screening but need to be linked to appropriate verification and follow-up care where required.

However, our scoping review also highlighted critical gaps in the literature, most importantly the scarcity of validity and cost-effectiveness assessments as well as research on outcomes other than infections. More robust evaluations of the identified methods are needed. In addition, future research may wish to consider how to further maximize the value of these additional contacts, for example, by reinforcing interventions promoting breastfeeding or uptake of family planning. Lastly, the implementation and scaling of post-discharge follow-up after childbirth will require investment and advocacy. Priority-setting exercises are, therefore, essential to ensure that new programs are affordable, meet local needs, and are supported by key stakeholders. Then, intensified follow-up of women and newborns after discharge from childbirth facilities has the potential to become an essential component of fostering a continuum of care for women and babies and of measuring and addressing postpartum morbidity.

**TABLE.** Overview of Included Studies on Follow-Up After Discharge From Childbirth Facilities

**Study**	**Study Location, Design, Population, and Sample Size**	**Study Outcome(s)**	**Data Collection Method, Person Making Contact, Timing of Postpartum Contact, and Persistence**	**Response**	**Other Comments**
**1.1. Studies using primarily home visits for follow-up**					
Aftab 2021^30^	Bangladesh, India, Pakistan, DRC, Ghana, Kenya, Tanzania, Zambia Prospective cohort of 125,716 pregnant individuals	Direct maternal morbidity and mortality, stillbirth, and neonatal death	Home visits by trained workers at week 1 and between week 7 and 11 after delivery; persistence not reported.	91% visited at least once	Study also included 3 home visits during pregnancy.
Creanga 2016^31^	Kenya Prospective cohort of 1,185 pregnant individuals	Intentions and behaviors regarding maternal and neonatal health service utilization	Home visits by trained interviewers up to week 6 after delivery; persistence not reported.	89%	Study also included 2 home visits during pregnancy.
Darmstadt 2010^32^	Bangladesh Cluster RCT of 10,006 neonates	Neonatal illness and survival	Home visits by CHWs at days 2, 5, 8, and 28 after delivery; persistence not reported.	73% visited at least once	Study included 2 home visits during pregnancy. CHWs attended the delivery if possible and facilitated referral if necessary.
Surkan 2017^33^	Bangladesh Prospective cohort within a cluster RCT of 59,666 pregnant individuals	Maternal morbidity and postpartum depression	Home visits by trained interviewers at months 3 and 6 after delivery; persistence not reported.	96% with depression data at 6 months	
Ward 2008^34^	United Kingdom Prospective cohort of 6,297 individuals with a CD	CD surgical site infection	Routine home visits by community midwives (median length of follow-up: 15 days after delivery); persistence not reported.	88% with completed follow-up records	
**1.2. Studies using primarily clinic visits for follow-up**					
Srun 2013^35^	Cambodia Prospective cohort of 222 individuals with a CD	CD superficial surgical site infection	Clinical assessment of wound by nurses and surgeons during inpatient stay and 2 scheduled clinic visits post-discharge at days 15 and 30 after delivery (microbiological methods used); phone follow-up by surgeons if patients didn’t return.	86% (Day 15) and 80% (Day 30) across all methods	9% (17/190) of those reached on day 15 were contacted by phone (on day 30: 16% (29/176)). 36% (4/11) of superficial infections were diagnosed post-discharge.
Zejnullahu 2019^36^	Kosovo Prospective cohort of 420 individuals with a CD	CD surgical site infection	Routine clinic visit (day 30) with gynecologists and additional outpatient department follow-up up to day 30 (microbiological methods used); persistence not reported.	77%	
**1.3. Studies using in-person visits for follow-up (location not specified)**					
Surveillance Monitoring for Antiretroviral Toxicities Yee 2021^37^	United States Prospective dynamic cohort (recruitment ongoing) of 2,976 pregnant individuals and individuals after delivery who are living with HIV (2007–2019)	Substance use in caregivers (wider study looks at health of children and their caregivers)	In-person structured interviews conducted by trained interviewers up to week 1 and at month 12 after delivery; persistence not reported.	98% (Week 1), 60% (Month 12)	
**2. Studies using primarily telephone calls for follow-up**					
Bianco 2013^38^	Italy Prospective cohort of 1,705 individuals after delivery	Postpartum infection	Telephone calls by 2 physicians (trained, not involved in patient care) at day 30 after discharge (medical records for validation); 5 attempts.	97%	Telephone surveillance identified more infections (8.9%) than traditional infection surveillance systems (1.4%).
Cardoso Del Monte 2010^39^	Brazil Prospective cohort of 204 individuals with a CD	CD surgical site infection	Telephone calls by study investigator and trained student nurse at days 15 and 30 after delivery; 3 attempts at each time point.	92%	
Hacker 2022^40^	United States Prospective cohort of 10,092 individuals after delivery	Hypertensive disorders	Telephone calls by a nurse or patient educator (and self-administered blood pressure measurement if cuff available) at week 1 after delivery; persistence not reported.	59%	
Halwani 2016^41^	United States Prospective cohort of 193 individuals with a CD	CD surgical site infection	Telephone calls by study investigator at days 7, 14, and 30 after delivery; 3 attempts at each time point.	82% interviewed at least once. 65% interviewed 3 times.	Incidence of infections detected by telephone 10% (19/193) compared to 7% (14/193) by traditional surveillance.
Hill 2021^42^	United States Prospective cohort of 631 individuals with a positive COVID-19 test during their hospital stay after delivery (individuals who tested negative were also included at 1 site)	Well-being of COVID-19 patients	Hospital records and telephone calls after discharge by physicians and clinical nurses up to week 2 after discharge (2 calls per week; first call within 3 days after discharge); persistence not reported.	36% reached a least once	
Lima 2016^43^	Brazil Prospective cohort of 528 individuals with a CD	CD surgical site infection	Telephone calls by trained undergraduate students up to day 15 and up to day 30 after delivery; 5 calls at each time point.	67% contacted at least once. 30% on day 15, 63% on day 30.	
Nguhuni 2017^44^	Tanzania Prospective cohort of 316 individuals with a CD	CD surgical site infection	Telephone calls by a clinically trained investigator or nurse at days 5, 12, 28 after delivery (clinical reviews for validation); at least 2 attempts.	87% reached at least once	85% of enrolled women provided a telephone number. Compared to clinical reviews, sensitivity and specificity of phone interviews was 72% and 100%, respectively.
PRAMS Salvesen von Essen 2022^45^	Puerto Rico Prospective cohort of individuals with a live birth: 1,536 (Phase 1), 1,485 (Phase 2)	Maternal and infant postpartum (ill-) health and behaviors (partly Zika-related)	Telephone calls by 6 interviewers at month 3 after delivery (Phase 1) and month 9 after delivery (Phase 2); persistence not reported.	77% (Phase 1), 83% (Phase 2)	Telephone surveys followed standard PRAMS protocol procedures.
Swissnoso SSI surveillance system Troillet 2017^46^	Switzerland Prospective cohort of 187,501 surgery patients including 32,814 individuals with a CD	Surgical site infection	Telephone calls by infection control nurses at 1 month after operation; 5 attempts.	91% for individuals with a CD	87% of CD surgical site infections diagnosed after discharge.
Woodd 2021^47^	Tanzania Prospective cohort of 879 individuals after delivery	Maternal postnatal infections and newborn infections	Telephone calls by research nurses (2 per hospital) at days 7 and 28 after delivery; 4 attempts over 7 days.	90% interviewed at least once. 86% interviewed on day 28.	3% of the initial sample had no access to a telephone.
**3.1. Studies using self-administered postal questionnaires for follow-up**					
The Norwegian Mother and Child Cohort Study Magnus 2016^48^ Bjelland 2016^49^	Norway Prospective cohort of 112,908 pregnant individuals	Maternal and child health	Self-administered postal questionnaire at month 6 after delivery; persistence not reported.	80% (Month 6)	Study also included additional questionnaires sent during pregnancy, at 18 months after delivery and later in childhood.
	NorwayProspective cohort of 20,248 pregnant individuals without pelvic pain in pregnancy			85% (Month 6)	
PRAMS Shulman 2018^50^ Kortsmit 2022^51^	United States Prospective cohort of individuals with a live birth (Annual state sample size: 1,000–3,000)	Maternal behaviors, attitudes, and experiences	Self-administered postal questionnaire with sampling taking place at months 2 to 6 after delivery; telephone follow-up for non-responders, 5 mailings and 15 call attempts.	47%–74% (median = 61%; in 2014)	
	United States Prospective cohort of 347,363 individuals with a live birth stratified by whether infant is alive or deceased			48.3% if infant is deceased, 56.2% if infant is alive	
Williams 2007^52^	United KingdomCross-sectional survey of 2,100 individuals after delivery	Perineal morbidity	Self-administered postal questionnaire at month 12; reminder after 3 weeks.	23%	
**3.2. Studies using self-administered electronic questionnaires for follow-up**					
Hirshberg 2018^53^	United StatesRCT of 206 individuals after delivery with pregnancy-related hypertension	Blood pressure monitoring	Text messages with individuals responding to automated text messages sent by web-based platform up to week 2 after discharge; 2 requests for blood pressure readings per day.	92% submitted at least 1 reading in the first 10 days postpartum	Texting reached more individuals than standard clinic visits (92% compared to 44%).
Zhu 2021^54^	ChinaProspective cohort of 674 individuals after delivery belonging to the rural-to-urban floating population	Self-efficacy, postpartum depression, and social support	Self-administered electronic questionnaire distributed via email or WeChat at weeks 6 and 12 after delivery; WeChat reminders 1 week and 1 day before data collection time points.	81% (Week 6), 65% (Week 12)	
**4. Studies using a combination of methods for follow-up**					
Baxter 2021^55^	EnglandRepeated point-prevalence study (4 time points) of 1,639 individuals with a CD	CD surgical site infection	Inpatient and re-admission cases identified via electronic records. Post-discharge cases identified via telephone and text messages (1st time point: telephone; 2nd–4th time point: text messages with telephone follow-up). Program led by midwife with infection control experience. Timing of contact not reported; 3 attempts for telephone calls.	47%–68% across all methods (1st time point: 60%, 2nd: 47%, 3rd: 68%, 4th: 60%)	Small quality improvement initiatives between time points. As accuracy of telephone numbers improved, response rates increased to 74%.
MINA-Brazil CohortCardoso 2020^56^Mosquera 2019^57^	BrazilProspective cohort of 1,246 pregnant individuals and individuals after delivery	Growth and development of Amazonian children	Linkage to hospital records, telephone calls (up to month 3), and study visits (from month 6 on) conducted by trained fieldworkers (including research assistants and nurses) on days 30 to 45, and months 3, 6 and 12 after delivery; multiple phone calls to schedule assessment and text message reminders before clinic visit.	64% (Month 6), 63% (Month 12)	Study also included visits during pregnancy, after delivery in hospital, and later during childhood (year 2 and planned for year 5).
	BrazilProspective cohort of 1,523 pregnant individuals and individuals after delivery			63% (Day 30–45)	3% of women did not provide valid telephone number.
Ferraro 2016^58^	ItalyProspective cohort of 3,685 individuals with a CD (4 time points)	CD surgical site infection	Routine clinic visit or telephone calls up to day 30 after delivery. Person making contact not reported; persistence not reported.	94% across all methods and time points	89% (129/145) of infections were diagnosed post-discharge.
Madhi 2018^59^	Panama, Dominican Republic, South Africa, MozambiqueProspective cohort of 3,243 pregnant individuals	Maternal and infant access to health care facilities	Visit to study site and, if necessary, telephone calls or home visits up to day 30. Person making contact not reported; persistence not reported.	98%	Study also included data collection at time of delivery.
Opøien 2007^60^	NorwayProspective cohort of 326 individuals with a CD	CD surgical site infection	Wound inspection in hospital by study authors, patients instructed to monitor symptoms and contact hospital, and self-administered postal questionnaire up to day 30 after delivery; reminders sent to non-responders and telephone follow-up if necessary.	100%	Incidence of infections detected by day 30 was 9% (29/326) compared to 2% detected before discharge.

Abbreviations: CD, cesarean delivery; CHW, community health worker; DRC, Democratic Republic of Congo; PRAMS, Pregnancy Risk Assessment Monitoring System; RCT, randomized controlled trial.

## Supplementary Material

GHSP-D-23-00377-supplement.pdf

## Notes

**Peer Reviewed**

**First Published Online:** April 9, 2024.
