# Supplementary material for: Current Approaches to Following Up Women and Newborns After Discharge From Childbirth Facilities: A Scoping Review
Source: Glob Health Sci Pract. 2024 Apr 29;12(2):e2300377. doi: 10.9745/GHSP-D-23-00377 (PMC11057794; doi:10.9745/GHSP-D-23-00377)
Supplement: GHSP-D-23-00377-supplement.pdf [file GHSP-D-23-00377-supplement.pdf]

Cite this article as: Pepper M, Campbell OMR, Woodd SL. Current approaches to following up women and newborns after discharge from childbirth facilities: a scoping review. *Glob Health Sci Pract*. 2024;12(2):32300377. https://doi.org/10.9745/GHSP-D-23-00377

Supplement. Search Strategy: Ovid MEDLINE(R) ALL, March 1, 2007 - November 2, 2022

#	**Query**
1	postpartum.mp. or Postpartum Period/
2	postnatal.mp. or Postnatal Care/
3	1 or 2
4	surveillance.mp. or Public Health Surveillance/ or Population Surveillance/
5	caesarean section.mp. or Cesarean Section/
6	3 or 5
7	4 and 6
